# Spontaneous Celiac Artery Dissection With Severe Pain Disproportionate to Lack of Prominent Tenderness and Limited Diagnostic Value of D-dimer and C-reactive Protein: A Case Report and Literature Review

**DOI:** 10.7759/cureus.88022

**Published:** 2025-07-15

**Authors:** Kenta Kanda, Yo Hattori, Shingo Suzuki

**Affiliations:** 1 Department of Internal Medicine, Chiba Central Medical Center, Chiba, JPN; 2 Department of Surgery, Chiba Central Medical Center, Chiba, JPN

**Keywords:** contrast-enhanced computed tomography (cect), c-reactive protein (crp), d-dimer levels, physical examination, spontaneous celiac artery dissection

## Abstract

Spontaneous celiac artery dissection (SCAD) typically presents with acute abdominal or back pain, although some patients are asymptomatic. We report a case of a 75-year-old woman who experienced sudden severe epigastric pain; SCAD was diagnosed using contrast-enhanced computed tomography (CT). Conservative management resolved her symptoms. A literature review of 256 SCAD cases, including ours, revealed that most cases exhibit a sudden or acute onset and severe pain disproportionate to mild or absent tenderness, with normal D-dimer and C-reactive protein levels. These findings highlight the importance of considering SCAD in cases of unexplained severe abdominal pain, warranting contrast-enhanced CT imaging even when the laboratory results are normal.

## Introduction

Spontaneous celiac artery dissection (SCAD) is a rare disease that typically presents with acute abdominal or back pain [1,2], although some cases may be asymptomatic [2-4]. While the pathogenesis remains unclear, potential risk factors include hypertension, smoking, and compression of the celiac trunk by the median arcuate ligament [2,3,5]. Contrast-enhanced computed tomography (CT) is crucial for diagnosis, and advancements in imaging modalities have led to an increase in reported SCAD cases [6], with some diagnosed incidentally [6,7]. However, there is no established diagnostic consensus, and the ambiguous symptoms can sometimes lead to misdiagnosis [8]. Studies suggest that most patients present with abdominal tenderness with no peritoneal irritation and negative D-dimer levels [9,10], which is similar to the classic presentation of acute mesenteric ischemia [6,11,12]. However, the sensitivity of the clinical presentations, physical examination findings, and laboratory test results, such as those of D-dimer and C-reactive protein (CRP) for SCAD, remains unclear. The management of SCAD depends on the presence of complications such as organ ischemia or aneurysm rupture. Conservative treatment comprising bowel rest, blood pressure management, and pain control is typically effective for uncomplicated cases [2,3,4,13]. Although some reviews have reported the possible efficacy of antiplatelet or anticoagulation therapy, their significant benefits remain unclear [3,4,8]. For cases with complications, interventions such as catheter-based treatments (stent placement or coil embolization) or surgical revascularization may be necessary [13,14].

Herein, we present a case report and literature review of the clinical features, physical examination, and laboratory test (D-dimer and CRP levels) results, and imaging findings for a definitive diagnosis of SCAD, as well as its treatment options.

## Case presentation

A 75-year-old woman presented to the emergency department of Chiba Central Medical Center, Japan, with sudden epigastric pain for two days. She also experienced acute, severe back pain three days before her visit. The pain was persistent and dull, rated 8/10 on the Numerical Rating Scale, and did not worsen with movement, although it progressively intensified. She denied experiencing nausea, vomiting, diarrhea, weight loss, or loss of appetite. Her medical and allergy histories were unremarkable, with no history of hypertension, diabetes, or cardiovascular disease. She had no known drug allergies. Family history was negative for vascular diseases or connective tissue disorders. Regarding lifestyle factors, she was a non-smoker with no history of alcohol abuse or recreational drug use. She was not taking any medications, including over-the-counter nonsteroidal anti-inflammatory drugs (NSAIDs) or hormonal supplements. Upon admission, the patient was afebrile, and the vital signs were normal. An abdominal examination revealed a soft and flat abdomen with no abnormal bowel sounds, tenderness, or peritoneal irritation. Laboratory tests performed immediately upon hospital arrival showed a white blood cell count of 9,620/µL, a CRP level of 7.35 mg/dL, and a D-dimer level of 0.6 µg/mL (Table 1).

**Table 1:** Results of the laboratory tests Alb, albumin; eGFR, estimated glomerular filtration rate; AST, aspartate aminotransferase; ALT, alanine aminotransferase; LDH, lactate dehydrogenase; G-GTP, gamma-glutamyl transpeptidase; CK, creatine kinase; ALP, alkaline phosphatase; CRP, C-reactive protein; CBC, complete blood count; WBC, white blood cell; RBC, red blood cell; Hb, hemoglobin; PT, prothrombin time; PT-INR, prothrombin time–international normalized ratio; APTT, activated partial thromboplastin time

Parameter	Patient values	Reference range
Serum chemistries		
Total protein	7.0	6.5-7.9
Alb (g/dL)	3.8	2.8-5.2
Urea (mg/dL)	12.7	8.0-20.0
Creatinine (mg/dL)	0.45	0.46-0.82
eGFR (ml/min/1.73m^2^)	99	-
Totalb bilirubin (mg/dL)	0.5	0.3-1.2
Direct bilirubin (mg/dL)	0.2	0.0-0.4
AST (U/L)	25	5-45
ALT (U/L)	24	10-40
LDH (U/L)	168	120-245
G-GTP (U/L)	21	0-48
CK (U/L)	40	50-210
Amylase (U/L)	83	39-134
ALP (U/L)	71	38-113
Electrolytes		
Na (mEq/L)	137	135-145
K (mEq/L)	3.7	3.5-5.0
Cl (mEq/L)	101	98-108
Ca (mg/dL)	9.0	8.6-10.2
P (mg/dL)	2.9	2.5-4.5
Inflammatory markers		
CRP (mg/dL)	7.35	0.00-0.30
Cardiac biomarkers		
Troponin T (ng/mL)	0.010	0.00-0.014
CBC		
WBC (/μL)	9620	3500-9700
Neutrophil (%)	62.0	40.6-76.4
Lymphocyte (%)	18.0	2.0-10.0
Monocyte (%)	19.0	2.0-10.0
Eosinophil (%)	1.0	0.0-8.5
Basophil (%)	0.0	0.0-3.5
RBC (×10^4^/μL)	408	376-516
Hb (g/dL)	12.0	11.2-15.2
Hematocrit (%)	36.4	34.3-45.2
Platelet (×10^4^/μL)	21.3	14.0-37.9
Coagulation studies		
PT (sec)	11.1	10.0-13.5
PT-INR	0.96	0.90-1.13
APTT (sec)	28.5	26-38.0
D-dimer (μg/mL)	0.6	0.0-1.0

**Figure 1:**
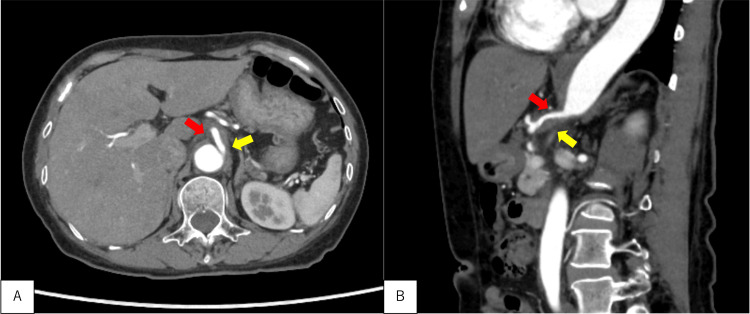
Contrast-enhanced CT images Contrast-enhanced CT (horizontal (A)/sagittal (B) plane) showing a thrombosed dissection of the celiac artery with narrowing of the luminal diameter; other major abdominal arteries remain intact (red arrow: true lumen, yellow arrow: false lumen) CT, computed tomography

An initial chest radiograph obtained within 30 minutes of arrival indicated an infiltrative shadow in the right lower lung field, whereas an abdominal ultrasound revealed no signs of aortic dissection, superior mesenteric artery dissection, or pancreatitis. Further imaging with contrast-enhanced CT, completed within 90 minutes of arrival, revealed increased fat density around the origin of the celiac artery and an infiltrative shadow in the right middle lobe. Contrast-enhanced CT also revealed thrombosed dissection of the celiac artery extending to the common hepatic artery with narrowing of the luminal diameter, while the other major abdominal arteries remained intact (Figure 1).

The final diagnoses were SCAD and acute bacterial pneumonia. Conservative management was selected because the patient showed no signs of ischemic complications and remained hemodynamically stable. Management involved bowel rest, pain control with non-steroidal anti-inflammatory drugs, blood pressure control, and antithrombotic therapy with aspirin, resulting in pain resolution by the third day. After confirming no exacerbation of abdominal pain for approximately one week following the resumption of oral food intake, we performed a follow-up CT on the ninth day, which confirmed no progression of the dissection, and the patient was subsequently discharged. Since the patient had no sputum symptoms and sputum culture could not be obtained, empirical antibiotic therapy with ampicillin/sulbactam was initiated. Acute bacterial pneumonia was diagnosed based on improvement of inflammatory markers following treatment and the resolution of the infiltrative shadow on the follow-up CT.

## Discussion

Review method

**Figure 2:**
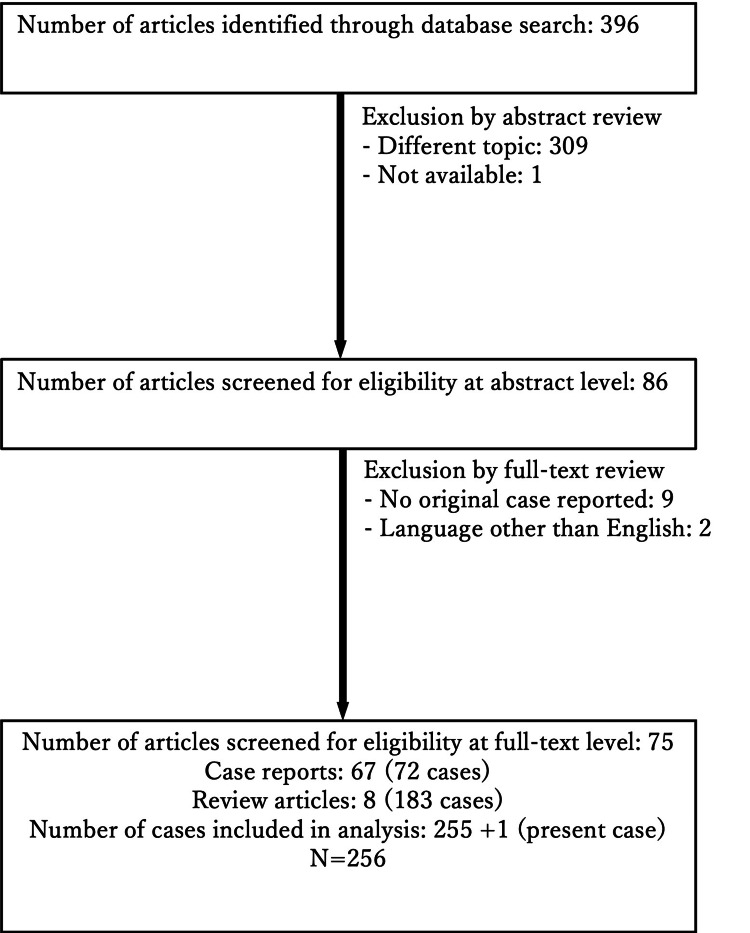
Flow diagram and search strategy for the literature review on spontaneous celiac artery dissection

We conducted a literature review using the PubMed database and searched for articles published between January 2004 and August 2024 using the keyword ‘celiac artery dissection’ to review the data. Original case reports and review articles were included in this analysis. The search results were screened by full-text review based on the following exclusion criteria: absence of original data for each case and articles not written in English. Figure 2 presents the screening process.

The following parameters were collected for analysis: sex; age; onset, location, and intensity of pain; signs of peritoneal irritation; intensity of tenderness; D-dimer level; CRP level; imaging for definitive diagnosis; and treatment option. A cut-off of <1.0 μg/mL [15] or, when a cut-off was not shown, a “positive/negative” description was considered for D-dimer; any article describing only the average was excluded. For CRP, a cut-off of <0.5 mg/dL [15-17] or, when a cut-off was not shown, a “positive/negative” description was considered. Severe pain was defined as pain with intensity, resistance to painkillers, or the need for opioids. Two unconscious patients with trauma were excluded from the pain analysis and physical examination. Patient data were analyzed using descriptive statistics.

Review results

Our search yielded 256 cases of SCAD (255 from 75 publications [3,4,7,9-12,14,18-84] plus the present case; Figure 2). The data from these SCAD cases are summarized in Table 2. The male-to-female ratio was 7:1.

**Table 2:** Results of the literature review on spontaneous celiac artery dissection Data compiled from 75 publications [3,4,7,11,12,14-83] and the present case CRP, C-reactive protein; CT, computed tomography; US, ultrasonography; MRI, magnetic resonance imaging

Variable	Value
Sex (N=256)	
Male	222 (86.7%)
Female	34 (13.3%)
Age (N=254)	
Mean age, years (range)	51.8 (18-82)
Onset (N=125)	
Sudden	69 (55.2%)
Acute	42 (33.6%)
Chronic	6 (4.8%)
Asymptomatic	8 (6.4%)
Chief complaint (N=202)	
Abdominal pain	143 (70.8%)
Back pain	27 (13.4%)
Abdominal pain with back pain	24 (11.9%)
Ill-defined pain	7 (3.5%)
Upper gastrointestinal bleeding	1 (0.5%)
Asymptomatic	24 (11.9%)
Severity of pain (N=61)	
Severe	51 (83.6%)
Mild	5 (8.2%)
No pain	5 (8.2%)
Intensity of tenderness (N=61)	
Severe tenderness	0 (0.0%)
Mild tenderness	47 (77.0%)
No tenderness	14 (23.0%)
Signs of peritoneal irritation (N=61)	
Present	1 (1.6%)
Absent	60 (98.4%)
Laboratory findings	
CRP (N=55)	
Negative	52 (94.5%)
Positive	3 (3.6%)
D-dimer (N=20)	
Negative	20 (100.0%)
Positive	0 (0.0%)
Imaging (N=256)	
Contrast-enhanced CT	253 (98,8%)
US	2 (0,8%)
MRI	1 (0.4%)
Treatment (N=255)	
Conservative	180 (70.6%)
With antiplatelet therapy	34 (13.3%)
With anticoagulation therapy	43 (16.9%)
No antiplatelet or anticoagulation therapy	92 (36.1%)
Endovascular treatment	69 (27.1%)
Surgery	6 (2.4%)

Insights from the literature review

Based on our review of reported cases meeting the defined inclusion criteria, middle-aged males comprised the majority of patients with SCAD, consistent with the general epidemiology of this condition [10]. Patients with SCAD presented with abdominal or back pain. The intensity of pain was often severe, and clinical presentation occurred at a sudden or acute onset. However, physical examination showed minimal or no tenderness. Our case, characterized by intense pain at sudden onset without prominent tenderness, strongly suggested a vascular issue rather than a typical intraabdominal organ problem [11,12].

D-dimer levels were negative in all patients. This suggests that the D-dimer test, although highly sensitive for aortic dissection [20], is irrelevant in SCAD diagnosis owing to its frequent negativity in these cases. Furthermore, most patients with SCAD were negative for CRP, whereas many patients with aortic dissection presented CRP positivity, providing insights into disease progression [21]. The pathophysiological difference likely relates to the smaller vessel size and different hemodynamic forces involved in celiac artery dissection compared with aortic dissection. Although aortic dissection creates extensive false lumens with significant activation of the coagulation cascade and fibrinolytic activity and intramural inflammation, celiac artery dissection involves a much smaller vascular territory with less activation. Only three CRP-positive cases have been reported, including ours, one of which was due to Behçet vasculitis. The high CRP level in our patient was due to pneumonia, highlighting that elevated CRP should not immediately direct suspicion toward SCAD complications when another infectious or inflammatory process is present. Contextual interpretation of inflammatory markers is crucial, as CRP elevation may reflect concurrent conditions rather than the vascular pathology itself. Almost all SCAD cases without complications were CRP-negative. There was no clear relationship between SCAD and pneumonia in the reviewed articles, suggesting that their co-occurrence was coincidental in our case.

Abdominal ultrasound is useful because of its noninvasive and straightforward nature. However, it is limited by its restricted imaging field and difficulty in assessing ischemia or detecting the precise location of the dissection [11,18]. Although plain abdominal CT can detect signs, such as arterial diameter enlargement and increased density of perivascular fat [10], its poor sensitivity makes it difficult to rule out SCAD in the absence of these specific findings. Therefore, the most definitive diagnostic tool for SCAD is a two-phase abdominal contrast-enhanced CT, as it allows visualization of arterial narrowing and fusiform irregularities [85,86]. Given the diagnostic challenges [6,10] and the need for radiologist involvement for CT interpretation, we recommend that clinicians and emergency physicians maintain high clinical suspicion for SCAD in patients presenting with severe abdominal or back pain disproportionate to lack of prominent tenderness. Early radiology consultation should be sought when CT findings are ambiguous or when clinical suspicion remains high despite initially unremarkable imaging. For diagnosis, it is essential to have sufficient experience in gathering medical history, in performing an abdominal physical examination to assess pre-test probability, and in interpreting CT scans.

In the present case, the patient responded well to conservative management, highlighting the importance of tailored treatment strategies based on individual patient needs. More than half of the patients showed favorable outcomes with conservative treatment. The absence of antiplatelet or anticoagulation therapy does not influence the occurrence of complications or the progression of dissection [4,8]; however, further investigation is needed to validate their efficacy and identify the specific cases in which their use would be most appropriate.

Limitations

This study had some limitations that warrant careful consideration. First, the study was limited by the inclusion of only reported cases and missing data, which raised a potential for selection bias. Future studies should focus on consecutively collected cases and minimize missing data to obtain reliable results. Furthermore, the absence of randomized controlled trials and large-scale observational studies weakened the ability to draw causal inferences from the data. Additionally, the lack of standardized data across case reports and review articles, particularly for subjective measures such as pain severity, introduced variability that may have compromised the reliability of the conclusions. These limitations underscore the need for a cautious interpretation of the results and highlight the need for further research to validate the findings and enhance our understanding of SCAD.

## Conclusions

SCAD is a rare and challenging diagnosis in abdominal emergencies. Sudden or acute severe abdominal pain disproportionate to mild or absent tenderness should raise the suspicion of underlying vascular pathology. Importantly, negative D-dimer or CRP results should not rule out celiac artery dissection, and early imaging remains essential in unexplained abdominal or back pain. Therefore, biphasic contrast-enhanced abdominal CT is crucial for establishing a definitive diagnosis when SCAD is suspected based on clinical information. Emergency Unit physicians and urgent care providers should maintain high clinical suspicion for SCAD in patients presenting with severe abdominal pain disproportionate to the lack of prominent tenderness and even normal inflammatory markers to ensure prompt diagnosis and appropriate management. Further accumulation of well-documented cases and studies is required to establish evidence-based diagnostic algorithms and optimal management strategies for SCAD.
